# Therapeutic effects of pleural and abdominal fluid filtration, concentration, and reinjection based on bioelectrical impedance analysis

**DOI:** 10.20407/fmj.2025-032

**Published:** 2026-05-14

**Authors:** Masanobu Usui, Norimasa Tsuzuki, Masutaka Tokuda, Akihiko Futamura, Miyo Murai, Akihiro Ito, Yoshikazu Koide

**Affiliations:** Department of Surgery and Palliative Medicine, Fujita Health University, School of Medicine, Toyoake, Aichi, Japan

**Keywords:** Refractory fluid retention, Thoracoabdominal fluid filtration concentration and re-injection, Bioelectrical impedance analysis, Body composition components

## Abstract

**Objective:**

The use of bioelectrical impedance analysis (BIA) has enabled measurement of the amounts of body components. Terminal cancer patients often experience excessive fluid retention. In this study, we investigated the effect of cell-free and concentrated ascites reinfusion therapy (CART) on fluid retention in terminal cancer patients using BIA.

**Methodology:**

Of the 676 patients, 176 underwent CART, with 6 patients having measured total body water (TBW), extracellular water (ECW) and body fluid balance (ECW/TBW) using BIA before and after CART.

**Results:**

Body composition measurements were obtained a total of 45 times before and after CART. The median (quartile) drained fluid volume by CART was 3000 mL (800–6000). After CART, the ECW/TBW improved to 0.413 (0.408–0.420) in patients with ascites. Correlation with pre-CART anthropometric analysis showed a positive correlation between water loss and TBW and ECW/TBW (R=0.415 and 0.528, P<0.001 and 0.001, respectively). A strong correlation was also observed between body weight and TBW and BCM (R=0.892 and 0.897, P<0.001 and 0.001, respectively). Furthermore, in cases with a large amount of fluid removal, BCM decreased by approximately 9 kg, from 32.3 kg to 23.2 kg. ECW/TBW, a measure of edema, was 0.465 before the start of the study, indicating severe fluid retention, but eventually improved to 0.404.

**Conclusion:**

In terminal cancer patients having refractory fluid retention, CART ameliorated edema. It was suggested that fluid retention may affect measurements of somatic cell volume, potentially hindering accurate assessment of these values.

## Introduction

Patients in the terminal stages of cancer often suffer from disease progression and cachexia as well as a decline in quality of life due to intractable fluid retention, such as generalized edema, pleural effusion, and ascites. Pleural effusion causes dyspnea, and ascites causes pain and discomfort due to abdominal distention, decreased food intake, and anorexia, leading to decreased ADL, suffering, and a shortened life expectancy. Treatment primarily involves administering diuretics and reducing fluid intake, but drainage is often performed in cases of massive accumulation of thoracoabdominal fluid, particularly when dyspnea, vomiting, and other gastrointestinal symptoms are present. According to the guidelines of the Japanese Society for Palliative Medicine, performing large-volume fluid removal at once is not recommended for patients because it can increase patient distress and increase the risk of electrolyte imbalances.^1^ In contrast, cell-free and concentrated ascites reinfusion therapy (CART) is a method in which ascites are filtered and concentrated then re-infused intravenously.^2^ Given the minimally invasive nature of the procedure, the expected reduction in nutrient loss due to fluid removal and ability to remove large amounts of body fluid at once, this is considered an effective treatment option for fluid retention caused by malnutrition.^3^

In addition, blood tests, bioelectrical impedance analysis (BIA), and similar methods are used to objectively assess nutritional status, and it is noteworthy that these nutritional indicators can be used to predict prognosis in the terminal stages of cancer and evaluate improvements in nutritional status.^4^ However, although muscle mass and somatic cell mass are considered important indicators of body composition in patients with terminal-stage cancer, these measurements may not accurately reflect actual conditions owing to excess fluid. In particular, the methods used for body composition analysis are sensitive to water content, and excess fluids may distort measurement values.^5^ Patients with refractory fluid retention may have their muscle mass and body cell mass overestimated due to excess fluid,^5^ and it is possible that body composition measurements are inaccurate, leading to apparent changes in body composition before and after CART. There have been no studies measuring body composition components before and after CART, raising questions about whether it is possible to accurately assess the edema status and diagnose sarcopenia in terminally ill patients with cancer.

In this study, we report an objective evaluation of refractory fluid retention using BIA before and after CART.

## Methods

Of 676 patients admitted to our department from January 2019 to July 2020, 176 underwent CART, with six patients having their body composition measured before and after CART. Body composition, including total body water (TBW), extracellular water (ECW), somatic cell mass (body cell mass: BCM), and body fluid balance (ECW/TBW), was analyzed using InBody S10^TM^ (InBody Co., Ltd., Seoul) the day before and the day after CART. The criteria for CART were defined as a predicted prognosis of more than one month, presence of symptoms caused by ascites or pleural effusion, and inability to alleviate these symptoms with conventional diuretics. Drainage with CART, which is performed to relieve symptoms, involves total ascites drainage. Albumin (Alb) and transthyretin (TTR) values were used as nutritional indices.^6^ No restrictions on food intake or water consumption were placed, and albumin administration was not performed. This study was approved by the Fujita Medical School Medical Research Ethics Review Committee (HM16-401). Statistical tests were performed using SPSS v28 (IBM Corp., Armonk, NY, USA); continuous variables were analyzed using the Mann–Whitney U test and categorical variables were analyzed using Fisher’s exact test, with P<0.05 considered statistically significant. Correlation was determined using Spearman’s rank correlation coefficient; P<0.05 indicated statistical significance and r≥0.4 or r≤–0.4 indicated correlation.

## Results

Of the six patients, two had hilar cholangiocarcinoma, two had lung adenocarcinoma, one had pancreatic head cancer, and one had gastric cancer. The median age of the patients (four males and two females) was 72.0 years (45–86). The median number of CART procedures was 10 (2–18), and the median number of drainage-only procedures was 2 (0–21). All deaths were confirmed by our department, and the median time from initial diagnosis to death was 150.5 days (67–1,566 days; Table 1). Median (quartile) serum Alb was 2.8 g/dL (2.1–3.5) and median (quartile) serum TTR was 12.9 mg/dL (6.7–35.9), with both median values being lower than the lower limit of the normal range. Fifty-nine CART procedures were performed, with 34 of these involving fluid removal only. Body composition measurements were obtained in a total of 45 cases, with a median (quartile) drained fluid volume of 3000 mL (800–6000) and median (quartile) perfusion volume of 250 mL (110–600) (Table 2).

The pre- and post-CART differences for each case are shown as Δ. The mean change before and after performing CART, performed multiple times in each case, revealed a decrease of 0.3–4.0 kg (median 2.8 kg) in body weight, and an improvement of 0.0003–0.009 (0.0045) in ECW/TBW (Table 3). In a comparison of pleural effusion and ascites, the median (quartile) volume of drainage was 1100 mL (950–1300) for pleural fluid and 3550 mL (2925–4687) for ascites, whereas the median (quartile) volume of perfusion was 180 mL (140–240) for pleural fluid and 300 mL (183–345) for ascites, with both drainage and perfusion volumes being significantly higher for ascites. Before CART, patients with ascites tended to be heavier at an average weight of 47.3 kg compared to 41.6 kg for patients with pleural effusion and had significantly higher fluid retention before CART, with a median ECW/TBW of 0.410 (0.408–0.412) in patients with pleural effusion versus 0.420 (0.413–0.427) in patients with ascites (P<0.001; Table 4). However, when comparing pleural effusion and ascites after CART, patients with ascites tended to be heavier with an average weight of 43.5 kg compared to 40.6 kg for patients with pleural effusion, whereas the ECW/TBW ratio was similar in patients with pleural effusion (median 0.407 [0.406–0.412]) and those with ascites (0.413 [0.408–0.420]), indicating no significant difference in fluid retention between the two groups, with both groups showing improvement (P=0.023; Table 5).

Correlation analysis of the amount of fluid removed and body composition prior to CART showed positive correlations between the amount of fluid removed and TBW and ECW/TBW (R=0.415 and 0.528; P<0.001 and 0.001, respectively). A strong correlation was also observed between body weight and TBW and BCM (R=0.892 and 897, P<0.001 and 0.001, respectively). A very strong correlation was observed, particularly between TBW and BCM (R=0.993, P<0.001; Table 6). After CART, no factors were found to correlate with the amount of fluid removed, but there was a strong correlation between body weight and TBW and BCM (R=0.890 and 0.897, P<0.001 and 0. 001, respectively). A very strong correlation was also found between TBW and BCM (R=0.993, P<0.001). After CART, no correlation with ECW/TBW was evident (Table 7).

**Figure 1:**
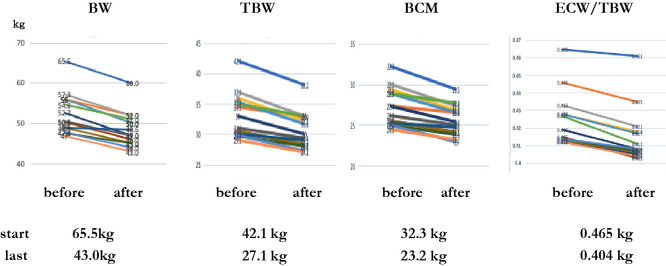
Changes in BIA data before and after CART in case 5 with massive ascites BW: body weight, TBW: total body water, BCM: basal cell mass, ECW/TBW: extra cellular water/TBW

Moreover, in the patient whom a large volume of fluid removal was performed, the patient’s weight before the first CART session (65.5 kg) decreased proportionally with the amount of fluid removed, and after the final CART session, it had decreased by more than 20 kg to 43 kg. Regarding weight loss, the amount of water decreased from 42.1 kg to 27.1 kg, indicating that the weight loss attributable to water was approximately only 15 kg, whereas BCM decreased from 32.3 kg to 23.2 kg, a reduction of approximately 9 kg. ECW/TBW was 0.465 before treatment, indicating severe fluid retention but eventually improved to 0.404 by the end of the study (Figure 1).

## Discussion

The most common cause of fluid retention is hypoalbuminemia, with treatment involving the administration of amino acids, which support protein synthesis; therefore, enteral or parenteral nutrition is considered effective.^7^ In contrast to albumin, TTR is primarily used clinically as an indicator of nutritional status. TTR is a blood protein with a short half-life of 1.9 days, making it ideal for reflecting changes in nutritional status over a short period of time, and was, therefore, used in this study to evaluate nutritional status.^6^ However, as protein synthesis is difficult in patients with terminal-stage cancer even when amino acids are administered, the addition of diuretics often contributes more to intravascular dehydration than to efficacy, and the median value of 12.9 mg/dL in this study was well below the lower limit of 22.0 mg/dL for normal values. Cachexia in patients with terminal cancer differs from simple starvation in that it is a disease characterized by resistance to nutritional management and treatment as well as significant muscle and weight loss associated with cancer progression. Nutritional assessment is crucial for understanding a person’s nutritional status, and in recent years, body composition analysis using BIA has been attracting attention. Using BIA, it is possible to measure indicators of sarcopenia, such as muscle mass, and indicators of edema, such as extracellular fluid ratio. In our department, we perform BIA using InBody S10TM to measure body composition, estimating it from electrical resistance measurements. This enables the following rapid, non-invasive measurements: the main measurements, which are expected to be abnormal in terminally ill patients with cancer, include: 1) measurements of body fat percentage and muscle mass, 2) detailed evaluations of the ratio of body fat to muscle, 3) measurements of body water content, 4) estimates of basal metabolic rate (BMR) and calculations of energy expenditure at rest, and 5) measurements of skeletal muscle mass. Although there have been reports on the usefulness of BIA as an indicator of sarcopenia in patients with advanced pancreatic cancer undergoing anticancer treatment,^8^ few reports have examined BIA as a prognostic factor for patients with terminal cancer who require palliative care, other than our report on pancreatic cancer.^9^ In particular, ECW/TBW is an excellent indicator of the degree of body edema. In general, ECW/TBW in a healthy population is approximately 0.38, and a person whose ECW/TBW exceeds 0.4 is defined as overhydrated.^10^ Zheng et al. reported on the usefulness of BIA method in patients with advanced cancer, specifically noting that an ECW/TBW of ≥0.40 is a risk factor for a poor prognosis.^11^ In diseases accompanied by edema, this value increases, primarily due to an increase in ECW. Furthermore, in cases of poor nutritional status due to aging or sarcopenia, intracellular water content decreases, leading to a higher ECW/TBW ratio. ECW/TBW is a good indicator of edema, but it is also widely used to assess nutritional status and disease severity. However, there have been no reports examining the usefulness of body composition analysis for disease-specific prognostic factors in patients with end-stage cancer, particularly those suffering from pancreatic cancer. In the present study, the median value for ascites accumulation was 0.420 (0.413–0.427), with many cases exceeding this value, which is considered indicative of severe edema. However, BCM was also high, particularly in patients with ascites, at 24.3 (17.4–26.1), with many cases not meeting the diagnostic criteria for sarcopenia^12^ proposed by the AWGS2019. Although body composition measurement using BIA can provide relatively accurate values in healthy individuals, in patients with kidney, liver, or heart disease who have edema or ascites, increased body water leads to an underestimation of the relative proportion of adipose tissue because water is relatively evenly distributed compared to adipose tissue. As a result, body fat percentage estimates tend to be too low,^5^ which can lead to overestimates of BCM and skeletal muscle mass, but may also indicate values different from actual muscle mass, especially in cases of overhydration or dehydration.^13^

Developed by Yamazaki et al. in 1973,^14^ CART is currently used in conditions such as liver cirrhosis and malignant ascites.^15^ CART is frequently used in treating terminally ill patients with cancer, with few medical institutions performing CART for refractory ascites in malnourished patents.^16^ Although there is global evidence of use of CART for hepatic ascites,^17–19^ malignant ascites is excluded from treatment due to concerns about dissemination, and there have been no reports of malignant ascites. Japanese guidelines for massive ascites do not recommend drainage of large volumes of fluid at once because rapid fluid volume fluctuation may increase patient discomfort and risk of electrolyte abnormalities,^1^ with similar views expressed in overseas reports; American palliative care guidelines^20^ recommend against large volume paracentesis, stating that it should be done carefully and incrementally as needed. In contrast, CART allows the removal of a large volume of ascites fluid at once, which may provide symptomatic relief. In this study, many patients were discharged the day after CART, so we were unable to analyze symptom changes using the assessment sheet because there was no record of symptoms the day after CART. However, prior studies on CART have mostly reported that it is effective in alleviating symptoms.^2,15,21^ Although CART is often used to treat ascites, we also use it to treat malignant pleural effusion. Pleural effusion can cause symptoms such as dyspnea, cough, and decreased exercise tolerance. When symptoms are difficult to control with oxygen, opioids, diuretics, and other medications, invasive drainage often provides improvement, but prolonged hospitalization and long-term treatment may be necessary. This can negatively impact the quality of life of terminally ill patients with cancer. In a previous study by our department, we reported results from 71 cases of pleural fluid CART performed on 29 patients with massive pleural effusions with the aim of reusing the albumin contained in the pleural fluid and alleviating clinical symptoms.^22^ The results before and after pleural effusion CART showed not only maintenance of nutritional status, but also significant improvement in clinical symptoms, such as dyspnea, depression, and insomnia.^18^ In our department, we perform pleural effusion drainage and CART for ascites, aiming to relieve symptoms, improve quality of life, and maintain nutritional status.

A comparison of patients with pleural effusion and those with ascites showed that those with ascites had higher body weight and significantly higher ECW/TBW, indicating greater fluid retention. The correlation between fluid volume removed and body composition analysis prior to CART showed positive correlations with TBW, BCM, and ECW/TBW. This indicates that fluid retention is associated with overestimates of body water content and edema as well as somatic cell volume and muscle mass.

Although this study did not show a significant difference in BCM before and after CART, in cases with substantial fluid removal, weight decreased proportionally to the amount of fluid removed. According to BIA results, this weight loss was due to a decrease in body water content and muscle mass. This suggests that the BIA method may be incorrectly measuring fluid retention as muscle mass. BIA estimates body water and muscle mass based on their close relationship, but in patients with fluid retention, increased extracellular fluid can lead to errors in muscle mass estimation. Regarding fluid retention and measurement deviations, in patients with fluid retention, increased extracellular fluid can lead BIA to misinterpret it as muscle mass. Specifically, this study found that even if actual muscle mass does not change, increased fluid volume tends to lead to overestimates of muscle mass. This can lead to bias in the assessment of patients’ actual muscle mass and body composition, potentially resulting in erroneous clinical judgments. This study revealed that the amount of water removed is related to body weight and body water and also correlates with BCM and BMR, suggesting that body composition measurements taken before CART may misclassify true sarcopenia cases as outside the normal range. Therefore, BIA measurement should be performed after fluid removal or CART, especially in patients with significant fluid retention; if this is not possible, muscle mass should be measured by CT or dual-energy X-ray absorptiometry (DEXA) instead of BIA.

Limitations of this study include: 1) unclear criteria for deciding whether to perform CART or only fluid removal and 2) lack of standardization of factors such as food and fluid intake before and after CART, intravenous fluid administration (amount and type), and use of diuretics. Evaluation of nutritional status was considered to be an area requiring future study because it is influenced by oral intake and the use of supplemental intravenous nutrition.

In patients with end-stage cancer with refractory fluid retention, CART alleviates edema, as indicated by changes in body composition. Fluid retention may influence measurements of somatic cell volume and BMR, potentially hindering accurate assessment of these parameters. Consequently, conducting BIA after CART may eliminate the confounding effects of fluid retention and enable more accurate measurement results.

## Figures and Tables

**Table 1:** Patient’s background and list

Case	Origin	Age	Gender	Times of CART	Survival time from ITT (months)
1	PHC with Abd. dissemination	62	male	2	52.2
2	PHC·Abd. dissemination	76	male	3	2.2
3	Lung adenocarcinoma with LN meta.	72	male	3	4.4
4	Lung adenocarcinoma with thoracic dissemination	83	female	8	4.9
5	Gastric cancer with Abd. dissemination	72	male	14	5.1
6	Gastric cancer with Abd. dissemination	45	female	15	34.7
		72*		10*	5.0*

PHC: Perihilar carcinoma, Abd.: Abdominal, LN: Lymph node *: median

**Table 2:** Summary of patient’s data undergoing CART

Demographics	n=6 (total 45 times)
Alb (g/dL) median (min–max)	2.8 (2.1–3.5)
TTR (mg/dL) median (min–max)	12.9 (6.7–35.9)
Times of CART	45
Drainage volume ml	3000 (800–6000)*
Reperfusion volume ml	250 (110–600)*
Days from first visit to death (min–max)	150.5 (67–1566)

Alb: Albumin, TTR: Transthyretin CART: cell free concentration ascites reperfusion therapy * data: median (25%–75%)

**Table 3:** Difference of values before and after CART

Case	Weight (Δ)	TBW (Δ)	BCM (Δ)	ECW/TBW (Δ)
1	3.0±1.0	0.0±0.6	0.2±0.4	0.005±0.000
2	2.6±2.7	0.0±0.6	0.4±0.6	0.0003±0.046
3	0.3±2.4	0.2±0.1	–1.4±3.0	0.003±0.002
4	1.5±0.6	0.4±0.6	0.5±0.1	0.004±0.002
5	4.0±1.6	2.4±1.2	1.5±0.9	0.009±0.003
6	3.4±0.4	0.1±1.2	–0.1±1.0	0.006±0.003

Δ: Deruta, Difference before and after CART TBW: total body water, BCM: basal cell mass, ECW/TBW: extra cellular water/TBW data was mean±standerd division

**Table 4:** Comparison between pleural effusion and ascites before CART

	pleural effusion (n=13)	ascites (n=32)	P-value
DV	1100 (950–1300)	3550 (2925–4687)	<0.001
RV	180 (140–240)	300 (183–345)	0.007
BW	41.6 (41.0–72.8)	47.3 (38.5–53.3)	0.079
TBW	21.8 (21.4–32.4)	29.1 (20.9–31.2)	0.861
BCN	18.5 (18.0–27.3)	24.3 (17.4–26.1)	0.625
ECW/TBW	0.410 (0.408–0.412)	0.420 (0.413–0.427)	<0.001

DV: drainage volume, RV: reperfusion volume, BW: body weight TBW: total body water, BCM: basal cell mass, ECW/TBW: extra cellular water/TBW median (25–75%)

**Table 5:** Comparison between pleural effusion and ascites after CART

	pleural effusion (n=13)	ascites (n=32)	P-value
Weight	40.6 (39.4–69.7)	43.5 (35.3–50.0)	0.075
TBW	21.2 (20.8–33.6)	27.2 (21.1–30.3)	0.950
BCM	18.0 (17.7–28.1)	23.1 (17.6–25.2)	0.643
BMR	994 (981–1349)	1167 (995–1256)	0.735
ECW/TBW	0.407 (0.406–0.412)	0.413 (0.408–0.420)	0.023
PA	4.20 (3.80–4.30)	4.05 (3.90–4.20)	0.277

TBW: total body water, BCM: basal cell mass, BMR: Basal metabolic rate, ECW/TBW: extra cellular water/TBW, PA: phase angle median (25–75%)

**Table 6:** Correlation between drainage volume and BIA before CART

	DV	Weight	TBW	BCM	ECW/TBW
DV	—	—	—	—	—
Weight	0.192	—	—	—	—
TBW	0.415**	0.892**	—	—	—
BCM	0.387**	0.897**	0.994**	—	—
ECW/TBW	0.528**	0.181	0.226*	0.132	—

DV: drainage volume, TBW: total body water, BCM: basal cell mass, ECW/TBW: extra cellular water/TBW Spearman test: ** Two-tailed significance at 1% level, * Two-tailed significance at 5% level

**Table 7:** Correlation between drainage volume and BIA after CART

	DV	Weight	TBW	BCM	ECW/TBW
DV	—	—	—	—	—
Weight	0.140	—	—	—	—
TBW	0.337*	0.890**	—	—	—
BCM	0.308*	0.897**	0.993**	—	—
ECW/TBW	0.216	0.180	0.236	0.160	—

DV: drainage volume, TBW: total body water, BCM: basal cell mass, ECW/TBW: extra cellular water/TBW Spearman test: ** Two-tailed significance at 1% level, * Two-tailed significance at 5% level

## Supplementary Material

PDF-Japanese
