# Supplementary material for: Therapeutic effects of pleural and abdominal fluid filtration, concentration, and reinjection based on bioelectrical impedance analysis
Source: Fujita Med J. 2026 May 14;12(3):231–5. doi: 10.20407/fmj.2025-032 (PMC13433081; doi:10.20407/fmj.2025-032)
Supplement: Supplementary file 1 — PDF-Japanese [file fmj-12-231_s1.pdf]

Short report
タイトル：生体電気インピーダンス分析法からみた胸腹水濾過濃縮再静注法の治療効果

ランニングタイトル：体組成からみた胸腹水濾過濃縮再静注法の治療効果

藤田医科大学医学部外科・緩和医療学講座
臼井正信, 都築則正, 徳田倍将, 二村昭彦, 村井美代, 伊藤彰博, 小出欣和

Usui Masanobu, MD, PhD, Tsuzuki Norimasa, MD, PhD, Tokuda Masutaka, MD, Ph D, Futamura Akihiko , PhD, Miyo Murai, MD, PhD, Ito Akihiro, MD, PhD, Yoshikazu Koide, MD, Ph D
Department of Surgery and Palliative Medicine, Fujita Health University, School of Medicine, Toyoake, Aichi, Japan
Original Article
Corresponding author: Usui Masanobu, MD, PhD
Department of Surgery and Palliative Medicine, Fujita Health University,
School of Medicine, 1-98 Dengakugakubo, Kutsukakecho, Toyoake, Aichi
470-1192, Japan
Tel: 0562-93-2111
E-mail: masanobu.usui@fujita-hu.ac.jp

【abstract】
目的：近年，生体電気インピーダンス分析(BIA)法を用いることにより，体を構成する成分量を測定することが可能となっている．終末期がん患者はしばしば過剰な体液貯留を来し，QOL に大きな影響を与える．当科では，難治性胸・腹水に対して胸・腹水濾過濃縮再静注法(CART)を積極的に導入している．今回，BIA 法による体組成成分の結果からみた終末期がん患者の体液貯留を中心とした治療効果について検討した．
方法：2019 年 1 月から 2020 年 7 月までに当科に入院した 676 例中 CART を 176 例に行い，うち CART 前後で体組成を測定した 6 例を対象とした．CART 施行前と施行翌日に InBody S10^{TM}を用いて体組成：体水分量(TBW)，細胞外水分量(ECW)，体細胞量(BCM)，体水分均衡(ECW/TBW)を測定した．
結果：6 例の年齢の中央値は 72.0 歳（45-86），男性4例，女性2例．原疾患は肝門部胆管癌2例，肺腺癌2例，膵頭部癌1例，胃癌1例であった．全例当科で死亡確認しており，初診から死亡までの期間の中央値は 150.5 日（67-1566 日）であった． CART 前後で体組成を測定できたのは計 45 回であった．CART の抜水量の中央値(四分位)は 3000ml(800-6000)，灌流量の中央値(四分位)は 250ml(110-600)であった．各症例で複数回行った CART 前後の変化量の平均値は、体重で 0.3-4.0（中央値 2.8）kg 減少、ECW/TBW で 0.0003-0.009（0.0045）減少していた．胸水と腹水の比較では，抜水量の中央値(四分位)は胸水が 1100ml(950-1300)，腹水が 3550ml(2925-4687)であり，腹水で有意に多かった． CART 前で ECW/TBW が胸水患者の中央値が 0.410 (0.408-0.412) であるのに対し腹水患者では 0.420 (0.413-0.427)と有意に体液貯留していた．CART 後に腹水患者で 0.413 (0.408-0.420)と改善していた． CART 前の体組成分析との相関では，抜水量と TBW, ECW/TBW との間に正の相関を認めた（R=0.415, 0.528, P<0.001, 0.001）．また体重と TBW, BCM との間に強い相関を認めた（R=0.892, 897, P<0.001, 0.001）． また，抜水量の多い症例でみると抜水量に比例するように CART 初回開始前の体重 65.5kg が回数を重ねるごとに減少し、最終の CART 後には 43kg まで 20kg以上減っていた．体重減少に対して水分量が 42.1kg から 27.1kg と減量分は約 15kg にとどまり，BCM が 32.3kg から 23.2kg と約 9kg 減っていた．浮腫の指標である ECW/TBW は開始前が 0.465 と重度の体液貯留を示していたが、最終的には 0.404 と改善していた．
結論：終末期がん患者の難治性体液貯留に対する CART は，浮腫が改善していることが体組成成分からも立証できた．体液貯留は、体細胞量や基礎代謝量の測定値に影響を与え、これらの値の正確な評価を妨げる要因となっている可能性が示唆された．そのため、CART 後に BIA を行うことで、体液貯留の影響を排除し、より正確な測定結果を得ることが可能となると考えられた．

Key words：難治性体液貯留、胸腹水濾過濃縮再静注法、生体電気インピーダンス分析、体組成成分

【序論】がん終末期の患者は，病勢の進行，悪液質の合併とともに，全身浮腫，胸水・腹水貯留など，難治性体液貯留により QOL の低下をきたすことも多い．胸水が貯留した場合は呼吸困難をきたし，腹水貯留は腹部膨満による痛み・不快感，食事摂取の低下や食欲不振をきたし ADL が低下する要因となり，短い余命が苦痛によって悩まされる．治療法としては第一に利尿剤の投与，輸液の減量などが行われるが，胸腹水が大量に貯留し，特に呼吸困難や嘔吐などの消化器症状を呈している場合にはドレナージが行われることが多い．日本緩和医療学会のガイドラインによると、急激な体液の変動が患者の苦痛を増加させる可能性や、電解質異常のリスクを高めるため患者に対して一度に大量の抜水を行うことは推奨されていない [1)]．これに対し，胸腹水濾過濃縮再静注法（cell-free and concentrated ascites reinfusion therapy: CART）は，腹水を濾過濃縮後に再静注する治療法であり [2)]，侵襲度の低さと抜水による栄養成分の損失の低減が見込めることから，一度に大量の体液を抜水できることもあり，低栄養を原因とする体液貯留の治療法として有効な選択肢と考えられる [3)]．

　また，栄養状態を客観的に評価する方法として，血液検査や生体インピーダンス法（bioelectrical impedance analysis: BIA）などの測定が実施されており，これら栄養指標の測定が，がん終末期の予後予測だけでなく，栄養改善における評価にも使用できることが注目されている [4)]．しかし，終末期がん患者では、体組成の評価において、筋肉量や体細胞量が重要な指標とされているが、水分過剰の影響によりこれらの測定値が実態を正確に反映しない可能性があり、特に、体組成分析に用いられる方法は、水分含有量に敏感であり、過剰な水分が測定値を歪めることが知られている [5)]．難治性体液貯留患者は，筋肉量や体細胞量など水分によって過大評価されている可能性があり [5)]，CART 前後で、体組成成分が変化したように誤測定されていることが考えられる．これまで体組成成分を CART の前後で測定した研究はなく，より正確に終末期がん患者の浮腫の状態やサルコペニアの診断ができるのかが問われる．

　今回，難治性体液貯留に対し，CART 前後で BIA による客観的評価を行ったので報告する．

【方法】2019 年 1 月から 2020 年 7 月までに当科に入院した 676 例中 CART を 176 例に行い，うち CART 前後で体組成を測定した 6 例を対象とした．CART 施行前と施行翌日に InBody S10^{TM}（InBody Co., Ltd. Seoul）を用いて体組成：体水分量(total body water: TBW)，細胞外水分量(extracellular water: ECW)，体細胞量(body cell mass: BCM)，体水分均衡(ECW/TBW)を測定した．CART の適応については予後1ヶ月以上と予測され、腹水・胸水による症状を伴っていて通常の利尿剤では症状緩和ができない場合とした．症状緩和のための抜水であり CART の抜水は、腹水を全量抜くこととしている．また，栄養指標として albumin(Alb)値と transthyretin(TTR)値を用いた [6)]．なお，食事摂取や飲水に制限は加えていない．またアルブミン投与は行っていない．本研究は，藤田医

科大学医学研究倫理審査委員会(HM16-401)にて承認されている. 統計学的検定は, SPSS ver28.(IBM Corp., Armonk, NY, USA)を用いて行い, 連続変数は, Mann-Whitney U testを, カテゴリー変数は, Fisher's exact testを用いて分析を行い, P<0.05を有意差ありと判定した. また, 相関関係は Spearman の相関係数を求め, P<0.05 を有意差あり, r≧0.4, r≦-0.4を相関関係ありと判定した.

【結果】

6 例の原疾患は肝門部胆管癌2例, 肺腺癌2例, 膵頭部癌1例, 胃がん1例であった. 年齢の中央値(四分位)は 72.0 歳(45-86)で, 男性4例, 女性2名. CART 回数の中央値は 10 回(2-18)でドレナージのみの回数は中央値 2 回(0-21)であった. 全例当科で死亡確認しており, 初診から死亡までの期間の中央値(四分位)は 150.5 日(67 -1566 日)であった(Table 1). 栄養評価として血清 Alb 値は中央値(四分位)で 2.8g/dL(2.1-3.5)で血清トランスサイレチン値は中央値(四分位)で 12.9mg/dL(6.7-35.9)と正常下限より中央値は低値であった. CART 回数は 59 回で抜水のみは 34 回で, このうち CART 前後で体組成を測定できたのは計 45 回であった. 45 回の CART で抜水量の中央値(四分位)は 3000ml(800-6000), 灌流量の中央値(四分位)は 250ml(110-600)であった(Table 2).

それぞれの症例の CART 前後の差をΔで示す. 各症例で複数回行った CART 前後の変化量の平均値は、体重で 0.3-4.0(中央値 2.8)kg 減少しており、ECW/TBW で 0.0003-0.009(0.0045)と改善していた (Table 3). 胸水と腹水の比較では, 抜水量の中央値(四分位)は胸水が 1100ml(950-1300), 腹水が 3550ml(2925-4687)であり, 灌流量の中央値(四分位)は, 胸水が 180ml(140-240), 腹水が 300ml(183-345)と抜水量, 灌流量ともに腹水で有意に多かった. 体重は, CART 前で胸水患者 41.6kg に対して腹水患者が 47.3kg と重い傾向にあった. CART 前で ECW/TBW が胸水患者の中央値が 0.410 (0.408-0.412)であるのに対し腹水患者では 0.420 (0.413-0.427)と有意に体液貯留していた(p<0.001) (Table 4). 一方, CART 後の胸水と腹水の比較では, 体重は, CART 後で胸水患者 40.6kg に対して腹水患者が 43.5kg と重い傾向にあった. ECW/TBW は胸水患者の中央値が 0.407 (0.406-0.412)であるのに対し, 腹水患者で 0.413 (0.408-0.420)と体液貯留は変わらなかったが両群ともに改善していた(p=0.023) (Table 5).

抜水量と CART 前の体組成分析との相関では, 抜水量と TBW, ECW/TBW との間に正の相関を認めた(R=0.415, 0.528, P<0.001, 0.001, respectively). また体重と TBW, BCM との間に強い相関を認めた(R=0.892, 897, P<0.001, 0.001, respectively). 特に TBW と BCM の間に非常に強い相関を認めた(R=0.993, P<0.001)(Table 6). CART 後では, 抜水量と相関を認める因子はなかったが、体重と TBW と BCM の間に強い相関を認めた(R=0.890, 0.897, P<0.001, 0.001). また TBW と BCM の間に非常に強い相関を認めた(R=0.993, P<0.001). CART 後では ECW/TBW と相関するものはなかった (Table 6).

また, 抜水量の多い症例でみると抜水量に比例するように CART 初回開始前の体重

65.5kg が回数を重ねるごとに減少し、最終の CART 後には 43kg まで 20kg以上減っていた. 体重減少に対して水分量が 42.1kg から 27.1kg と減量分は約 15kg にとどまり, BCM が 32.3kg から 23.2kg と約 9kg 減っていた. 浮腫の指標である ECW/TBW は開始前が 0.465 と重度の体液貯留を示していたが、最終的には 0.404 と改善していた(Figure 1).

【考察】

体液貯留の原因として, 低アルブミン血症が最も多く, これに対する治療としては, アミノ酸の投与が蛋白質合成に有効であることから, 経腸または静脈栄養法が有効であるとしている [7] . アルブミンに対し、トランスサイレチン(Transthyretin, TTR)は、主に栄養状態の指標として臨床的に用いられる. 血中半減期が 1.9 日と短いため短期間での栄養状態変化を反映しやすい血中タンパク質であり、今回栄養状態の評価として用いた [6] . しかし, 終末期がん患者ではアミノ酸投与によっても蛋白合成が困難であるため, これに利尿剤を加えても効果よりも血管内脱水を助長するのみであることが多く、今回の検討でも中央値 12.9mg/dL と正常値の下限 22.0mg/dL を大きく下回っていた. 終末期がん患者の悪液質は, 単純な飢餓状態とは異なり, 栄養管理や治療に抵抗し, がんの進行に伴う著しい筋肉減少と体重減少を主徴とする疾患である. 栄養状態の把握にはアセスメントが重要であり, 近年, BIA による体組成分析が着目されている. BIA を用いることにより, サルコペニアの指標である筋肉量, 浮腫の指標である細胞外水分比などの測定が可能である. 当科では BIA を InBody S10™を用いて測定している. InBody はインピーダンス法による測定を行い、電気抵抗値から体組成を推定する. これにより、以下の非侵襲かつ迅速な測定が可能である. 主な測定項目としては、1)体脂肪率と筋肉量の測定、2)体脂肪と筋肉の割合を詳細に評価. 3)体水分量の測定. 4)基礎代謝量(BMR)を推定し安静時に消費されるエネルギー量を算出. 5)骨格筋量の測定. 終末期がん患者において, これらの測定値は異常となることが予想される. 抗がん治療を行っている進行膵がん患者のサルコペニア指標としての BIA の有用性の報告 [8]はみられるが, 緩和医療が必要な終末期がん患者の予後予測因子に関し, BIA を検討した報告はほとんど無く, 膵癌について我々が報告した [9]. 特に ECW/TBW は, 体の浮腫の程度を示す優れた指標である. 一般に, 健康な集団の ECW/TBW は, 約 0.38 であり, ECW/TBW が 0.4 を超える人は, 水分過剰と定義される [10]. Zheng らは, 進行がん患者における BIA 法の有用性, 特に ECW/TBW≧ 0.40 が予後不良の危険因子であると報告している [11] . 浮腫を伴う疾患がある場合, 主に ECW が増える形でこの数値が高くなる. さらに, 加齢・サルコペニアなど栄養状態が悪化した場合は, 細胞内水分量(Intracellular Water) が減少するため, ECW/TBW が高くなる. ECW/TBW は浮腫の指標でありながら, 栄養状態や疾患の重症度を示す指標として広く使用されている. しかし, 終末期がん患者, 特に膵がんのように疾患を特定した予後規定因子に関する体組成分析の有用性を検討した報告は見当たらない. 今回の検討でも特に腹水貯留では中央値が 0.420 (0.413-0.427)と重度浮腫とさ

れる 0.420 を超える症例が多かった. しかし, BCM も特に腹水患者で 24.3 (17.4-26.1)と高く, AWGS2019 が提唱するサルコペニアの診断基準 [12]を満たさない症例が多い. BIA 法を用いた身体組成測定は, 健常人においては比較的正確な値を得ることが可能であるが, 浮腫や腹水を有する腎・肝・心疾患患者などでは体水分が増加すると、脂肪組織と比較して水分は比較的均一に分布しているため、脂肪組織の相対的割合が低く見積もられる. 結果として、脂肪率が実際よりも低く評価される傾向があり [5]、%脂肪率、っm，、、、が過小評価されやすく, このため BCM や骨格筋量を過大評価につながるだけでなく、特に水分過剰や脱水状態では、実際の筋肉量と異なる値を示すことがある[13].

CART は 1973 年に山崎らによって開発され [14], 現在では肝硬変やがん性腹水などで実施されている [15]. CART は、終末期がん患者に対する治療が多く, 低栄養患者の難治性腹水に対して CART を実施している医療機関は少ない [16]. 海外において肝性腹水に対する CART の RCT を用いたエビデンスは存在するが [17-19]、悪性腹水は播種の懸念があることで治療対象外となり悪性腹水の報告はない. 大量腹水に対する本邦のガイドラインでは、一度に大量の抜水を行うことは急激な体液の変動が患者の苦痛を増加させる可能性や、電解質異常のリスクを高める可能性があり推奨されておらず[1]、また海外の報告においても、同様の見解が示されておりアメリカの緩和ケアガイドライン [20]では、大量の抜水は避けるべきとされており、必要に応じて少量ずつ慎重に行うことが推奨されている. これに対して CART は一度に大量の腹水を抜水することが可能で、これにより症状緩和も期待できる. 今回の検討では、CART 入院のため翌日退院が多く、アセスメントシートを用いた症状変化について CART 翌日の記載がなく科学的根拠となるデータが無いため検討できていないが、これまでの CART の論文では症状緩和ができたという報告が多い[2, 15, 21]. 一方 CART は腹水に対する治療を示すことが多いが, 当科では悪性胸水についても積極的に CART を行っている. 胸水貯留は呼吸困難, 咳嗽, 運動耐容能の低下などの症状を来し, 症状緩和のため酸素やオピオイドの投与, 利尿薬などの薬物療法でコントロールが困難な場合, 侵襲的ドレナージにより改善することが多く, 入院治療の延長や長期間の治療を余儀なくされる場合があり, 予後が限られた終末期がん患者にとって QOL を大きく損なうことになる. 当科の先行研究で 29 例の大量胸水貯留患者に対し, 胸水中に含まれる Alb の再利用や臨床症状の改善を目的に 71 回の胸水 CART を行った結果を報告した [22]. その結果, 胸水 CART 前後の結果は, 栄養状態の維持だけでなく, 臨床症状では呼吸困難, 気分の落ち込み, 不眠などが有意に改善していた [18]. 当科では症状緩和, QOL の改善だけでなく栄養状態の保持も目的として, 腹水に対する CART と同様, 胸水に対しても濾過濃縮再静注法を行っている.

胸水と腹水の比較では, 腹水患者のほうが体重が重く, ECW/TBW が有意に高くより体液貯留が高度であることが示された. 抜水量と CART 前の体組成分析との相関では, 抜水量と TBW, BCM, ECW/TBW との間に正の相関を認めた. このことは体液貯留量は体水分量や浮腫だけでなく体細胞量や筋肉量の過大評価つながっていることを意味し

ている.

今回の検討で CART 前後で BCM に有意な差は出なかったが, 抜水量の多い症例でみると抜水量に比例するように体重が減っていた. この減量分は BIA では体水分量だけではなく筋肉量も同様に減っていた. このため BIA 法において体液貯留を筋肉量と誤測定してしまっている可能性が示唆された. BIA は、体水分と筋肉量を密接に関連付けて推定するが、特に体液貯留患者では、細胞外水分が増加し、これが筋肉量の推定値に誤差をもたらす原因となっている. 体液貯留と測定結果の偏位については、体液貯留患者では、細胞外水分の増加により、BIA は水分を筋肉量と誤認識しやすくなる. 具体的には、実際の筋肉量が変化していなくても、水分の増加により筋肉量の推定値が過大になる傾向があり、今回の検討で明らかとなった. これにより、患者の実際の筋肉量や体組成の評価に偏りが生じ、誤った臨床判断につながる可能性が残る. 今回の検討で抜水量は体重や体水分に関与しているだけでなく体細胞量や基礎代謝量とも相関を認め, CART 前の体組成は, 水分も筋肉量とみなしていることから, 本来のサルコペニア症例を基準外と判定してしまっている可能性が示唆された. このため, 特に大量の体液貯留患者においては BIA 測定は抜水や CART の処置後に行うべきであり, できない場合の筋肉量の測定は BIA 法ではなく, CT や二重エネルギーX 線吸収測定法(DEXA)法を用いて行うべきであると考えられた.

本研究の限界としては、①CART を施行するか、ドレナージのみとするかの適応基準が不明確であったこと、②CART 前後の飲食量、点滴（量・種類）、利尿薬投与の有無など一定になっていない. 栄養状態の評価は、経口摂取量や静脈栄養併用の状況に影響されるため、今後の検討課題と考えられた。

【結論】終末期がん患者の難治性体液貯留に対する CART は, 浮腫が改善していることが体組成成分からも立証できた. 体液貯留は、体細胞量や基礎代謝量の測定値に影響を与え、これらの値の正確な評価を妨げる要因となっている可能性が示唆された. そのため、CART 後に BIA を行うことで、体液貯留の影響を排除し、より正確な測定結果を得ることが可能となると考えられた.

Acknowledgements
We would like to thank Dr. Akiko Kada (Associate Professor), Dr. Takuma Ishihara (Assistant Lecturer),and Dr. Yuki Matsuda (Assistant Professor) for their valuable guidance on the statistical analysis

著者に COI はありません.

【参考文献】

1) Japanese Society for Palliative Medicine. Clinical Guidelines for Gastrointestinal Symptoms in Cancer Patients. Tokyo Japan:KANEHARA & Co., Ltd; 2017:93.

2) Ito T, Hanafusa N. CART: Cell-free and Concentrated Ascites Reinfusion Therapy against malignancy-related ascites. Transfus Apher Sci 2017;56:703-7.

3) Chen H, Ishihara M, Horita N, Tanzawa S, Kazahari H, Ochiai R, Sakamoto T, Honda T, Ichikawa Y, Watanabe K, Seki N. Effectiveness of Cell-Free and Concentrated Ascites Reinfusion Therapy in the Treatment of Malignancy-Related Ascites: A Systematic Review and Meta-Analysis. Cancers (Basel) 2021;13:4873.

4) Crawford GB, Robinson JA, Hunt RW, Piller NB, Esterman A. Estimating survival in patients with cancer receiving palliative care: is analysis of body composition using bioimpedance helpful? J Palliat Med 2009;12:1009-14.

5) Kyle UG, Bosaeus I, De Lorenzo AD, Deurenberg P, Elia M, Gómez JM, Heitmann BL, Kent-Smith L, Melchior JC, Pirlich M, Scharfetter H, Schols J, Pichard C; Composition of the ESPEN Working Group. Bioelectrical impedance analysis—part I: review of principles and methods. Clinical Nutrition 2004; 23:1226-43.

6) Beck FK, Rosenthal TC. Prealbumin: A marker for nutritional evaluation. Am Fam Physician. 2002;65(8):1575-1578.

7) Muscaritoli M, Arends J, Bachmann P, et al. ESPEN practical guideline: Clinical Nutrition in cancer. Clin Nutr 2021;40: 2898-913.

8) Tozuka Y, Ueno M, Kobayashi S, Morimoto M, Fukushima T, Sano Y, Kawano K, Hanaoka A, Tezuka S, Asama H, Moriya S, Morinaga S, Ohkawa S, Maeda S. Prognostic significance of sarcopenia as determined by bioelectrical impedance analysis in patients with advanced pancreatic cancer receiving gemcitabine plus nab-paclitaxel: A retrospective study. Oncol Lett 2022;24:375.

9) Ito A, Usui M, Murai M, Tsuzuki1 M, Futamura A ,Imai K, Itani K. Investigation of prognostic factors in patients with terminal pancreatic cancer: Focus on clinical symptoms, cachexia-related proteins, and body composition analysis at admission. Fujita Med J 2025(in press).

10) Kyle UG, Bosaeus I, De Lorenzo AD, Deurenberg P, Elia M, Gómez JM, Heitmann BL, Kent-Smith L, Melchior JC, Pirlich M, Scharfetter H, Schols AM, Pichard C; Composition of the ESPEN Working Group.Bioelectrical impedance analysis--part II: utilization in clinical practice. Clin Nutr 2004;23:1430-53.

11) Zheng K, Lu J, Liu X, Ji W, Liu P, Cui J, Li W.The clinical application value of the extracellular-water-to-total-body-water ratio obtained by bioelectrical impedance analysis in people with advanced cancer. Nutrition 2022;96:111567.

12) Chen LK, Woo J, Assantachai P, et al. Asian Working Group for Sarcopenia: 2019 Consensus Update on Sarcopenia Diagnosis and Treatment. J Am Med Dir Assoc 2020;21:300-7.
13) Barbosa-Silva MCG, Barros AJD. Bioelectrical impedance analysis in clinical practice. Curr Opin Clin Nutr Metab Care 2005; 8：311-7.
14) Yamazaki Z. Fukusui no roka jyokin jyogan saibou nousyuku fukusui saichunyu ryouhou. Surgery 1975;37:1628-9 (in Japanese).
15) Hanafusa N, Isoai A, Ishihara T, et al. Safety and efficacy of cell-free and concentrated ascites reinfusion therapy (CART) in refractory ascites: post-marketing surveillance results. PLoS One 2017;12:e0177303.
16) Sawada Y, Nomura Y, Yoshii Y. Cell-free and concentrated pleural effusion reinfusion therapy for postoperative chylothorax. Jpn J Cardiovasc Surg Japanese Journal of Cardiovascular Surgery 2009;38:205-7 (in Japanese).
17) Smart HL, Triger DR. A randomised prospective trial comparing daily paracentesis and intravenous albumin with recirculation in diuretic refractory ascites. J Hepatol 10(2):191-7.1990
18) Bruno S, Borzio M, Romagnoni M, Battezzati PM, Rossi S, Chiesa A, Podda M. Comparison of spontaneous ascites filtration and reinfusion with total paracentesis with intravenous albumin infusion in cirrhotic patients with tense ascites. BMJ. 27;304(6843):1655-8.1992
19) Graziotto A, Rossaro L, Inturri P, Salvagnini M. Reinfusion of concentrated ascitic fluid versus total paracentesis. A randomized prospective trial. Dig Dis Sci. 42(8):1708-14, 1997.
20) National Consensus Project for Quality Palliative Care, 2018. Clinical Practice Guidelines for Quality Palliative Care. 2nd Edition.
21) Hanada R, Yokomichi N, Kato C, Miki K, Oyama S, Morita T, Kawahara R. Efficacy and safety of reinfusion of concentrated ascitic fluid for malignant ascites: a concept-proof study. Support Care Cancer. 2018.26(5):1489-1497.
22) Fujisaki H, Usui M, Ito A, Murai M, Tsuzuki1 M, Futamura A, Imai K, Itani K. Usefulness of Cell-Free and Concentrated Pleural Effusion Reinfusion Therapy in Terminal Cancer Patients. Fujita Med J 2025 (in press).

legend for figures

Table 1 Patient‘s background and list

Table 2 Summary of patient’s data undergoing CART

Table 3 Difference of values before and after CART

Table 4 Comparison between pleural effusion and ascites before CART
Table 5 Comparison between pleural effusion and ascites after CART
Table 6 Correlation between drainage volume and BIA before CART
Table 7 Correlation between drainage volume and BIA after CART
Figure 1 Changes in BIA data before and after CART in the case 5 with massive ascites
